# Contrast echocardiography: a practical guideline from the British Society of Echocardiography

**DOI:** 10.1186/s44156-023-00034-9

**Published:** 2023-11-15

**Authors:** Reinette Hampson, Roxy Senior, Liam Ring, Shaun Robinson, Daniel X. Augustine, Harald Becher, Natasha Anderson, James Willis, Badrinathan Chandrasekaran, Attila Kardos, Anjana Siva, Paul Leeson, Bushra S. Rana, Navtej Chahal, David Oxborough

**Affiliations:** 1 London North West University Healthcare NHS Trust, London, UK; 2 Royal Brompton Hospital and Imperial College, London, UK; 3 West Suffolk Hospital NHS Foundation Trust, Bury St Edmunds, UK; 4 Imperial College Healthcare NHS Trust, London, UK; 5 Royal United Hospitals Bath NHS Foundation Trust, Bath, UK; 6 Department for, Health University of Bath, Bath, UK; 7 Alberta Heart Institute, University of Alberta Hospital, Edmonton, Canada; 8 Warrington and Halton Teaching Hospital NHS Foundation Trust, Warrington, UK; 9 Wiltshire Cardiac Centre, Great Western Hospital NHS Trust, Swindon, UK; 10 Translational Cardiovascular Research Group, Department of Cardiology, Milton Keynes University Hospital, Milton Keynes, UK; 11 Faculty of Medicine and Health Sciences, University of Buckingham, Buckingham, UK; 12 Queen Alexandra Hospital, Portsmouth, UK; 13 Division of Cardiovascular Medicine, Radcliffe Department of Medicine, University of Oxford, Oxford, UK; 14 Liverpool Centre for Cardiovascular Science, Liverpool John Moores University, Liverpool, UK

**Keywords:** Ultrasound contrast agents, Echocardiography, Guideline

## Abstract

Ultrasound contrast agents (UCAs) have a well-established role in clinical cardiology. Contrast echocardiography has evolved into a routine technique through the establishment of contrast protocols, an excellent safety profile, and clinical guidelines which highlight the incremental prognostic utility of contrast enhanced echocardiography. This document aims to provide practical guidance on the safe and effective use of contrast; reviews the role of individual staff groups; and training requirements to facilitate its routine use in the echocardiography laboratory.

**Supplementary Information:**

The online version contains supplementary material available at 10.1186/s44156-023-00034-9.

## Introduction

Ultrasound contrast agent (UCA) has been well established as a safe, cost-effective, and reliable imaging tool for detecting heart disease [1–3]. UCA is an essential component of echocardiography laboratories, it reduces the need for additional downstream testing, provides results in real time, and increases workflows with a direct impact on patient outcomes [4]. This document aims to guide the echocardiographer in all aspects of UCA, ensuring the safe and efficacious use, ultimately to improve patient outcomes.

## Contrast agents: an overview

### Key points

1. There are  three commercially available contrast agents in the UK (SonoVue, Luminity, Optison), all of which contain high-molecular weight gas surrounded by an elastic shell.
2. They are 1-4 micron in size and can traverse both pulmonary and systemic micro vasculature.
3. There are differences between the agents in storage, preparation, and administration.
4. They may be administered as a slow bolus or intravenous infusion.
5. A dedicated infusion pump (Vuejet) is available for continuous infusion of SonoVue.

At present there are three commercially available ultrasound contrast agents (UCAs) in the United Kingdom which share some common properties; they are microbubbles containing inert bio-compatible high-molecular weight gas and are encapsulated within a shell (Table 1). Using high molecular weight gases with relatively low solubility and diffusivity limits, the outward diffusion of gas from its core and the encapsulating shell further reduces outward diffusion, improving stability after intravenous injection [5]. UCAs are sufficiently small (< 7 µm, size of the red blood cell) to pass unhindered through the pulmonary and systemic capillary beds and remains entirely intravascular [6].

**Table 1:** Commercially available ultrasound contrast agents in the UK

Name	Manufacturer	Shell	Gas
SonoVue	Bracco Diagnostics	Phospholipid	Sulphur hexafluoride
Luminity	Lantheus Medical Imaging	Phospholipid	Perflutren
Optison	GE Healthcare	Human albumin	Perflutren

All three contrast agents in Table 1 are approved only for intravenous use. Each contrast agent has specific storage conditions, shelf life and methods of preparation which are further described below.

## Contrast administration

### SonoVue

#### Storage

The product does not require any special storage conditions. Once reconstituted, chemical and physical stability has been demonstrated for 6 h, however it is recommended that the product be used immediately once prepared [7].

#### Preparation

Before use, examine the product to ensure that the container and closure have not been damaged. SonoVue is prepared by injecting 5 mL of normal saline into the contents of the vial. The vial is then shaken vigorously for at least twenty seconds until the lyophilisate is completely dissolved. The desired volume of the dispersion can be drawn into a syringe any time up to six hours after reconstitution. Do not use if the liquid obtained is clear and/or if solid parts of the lyophilisate are seen in the suspension [7]. It is important to look before administration to check that the liquid is milky white, this ensures no significant microbubble degeneration. This applies to all UCAs.

#### Bolus injection

Just before drawing the dispersion into a syringe, the vial should be agitated to re-suspend the microbubbles, SonoVue should be administered immediately after drawing into a syringe by injection into a peripheral vein. If SonoVue is not used immediately after reconstitution, the microbubble dispersion should be shaken again before being drawn up into a syringe. The recommended dose of SonoVue for cardiac chambers at rest or with stress is 2 ml, followed by a 5 ml flush of saline. However, the clinical experience endorsed by international guidelines is to use only 0.5 ml slow bolus at rest and stress which usually suffices as the latest machine technology is very sensitive in detecting the microbubbles. This can be repeated as required.

#### Continuous infusion

**Fig. 1:**
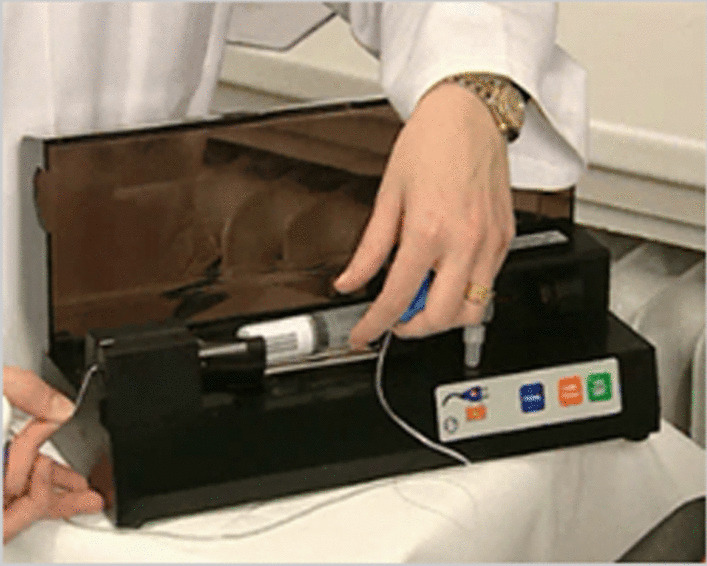
Vueject® pump produced by Bracco for continuous SonoVue infusion

The manufacturers of SonoVue produce a specific pump (Vueject®) (Fig. 1) which continuously oscillates and agitates the suspension, preventing separation of the contents. SonoVue is aspirated from the vial into a product specific 20 ml syringe, which is then placed inside the pump with an infusion line connected to the syringe. However, SonoVue can also be administered 1:1 diluted with saline. During initiation of the pump, it oscillates for 90 s initially as part of the setup, whereafter the pump continues to oscillate after initial mixing is complete. The infusion rate can be controlled via a touchpad and increased/decreased as necessary; as the rate is changed, the time remaining for contrast use is displayed simultaneously [8]. Typically, an infusion rate of 0.8–1.0 ml/min is optimal for both left ventricular opacification (LVO) and myocardial opacification.

### Luminity

#### Storage

The product has a shelf-life of two years and should be stored in a refrigerator between 2 and 8°C. Once activated it should be used within 12 h, if not, the product can be re-activated up to 48 h after initial activation and used up to 12 h after the second activation. After activation it should be stored below 30 °C [9].

#### Preparation

**Fig. 2:**
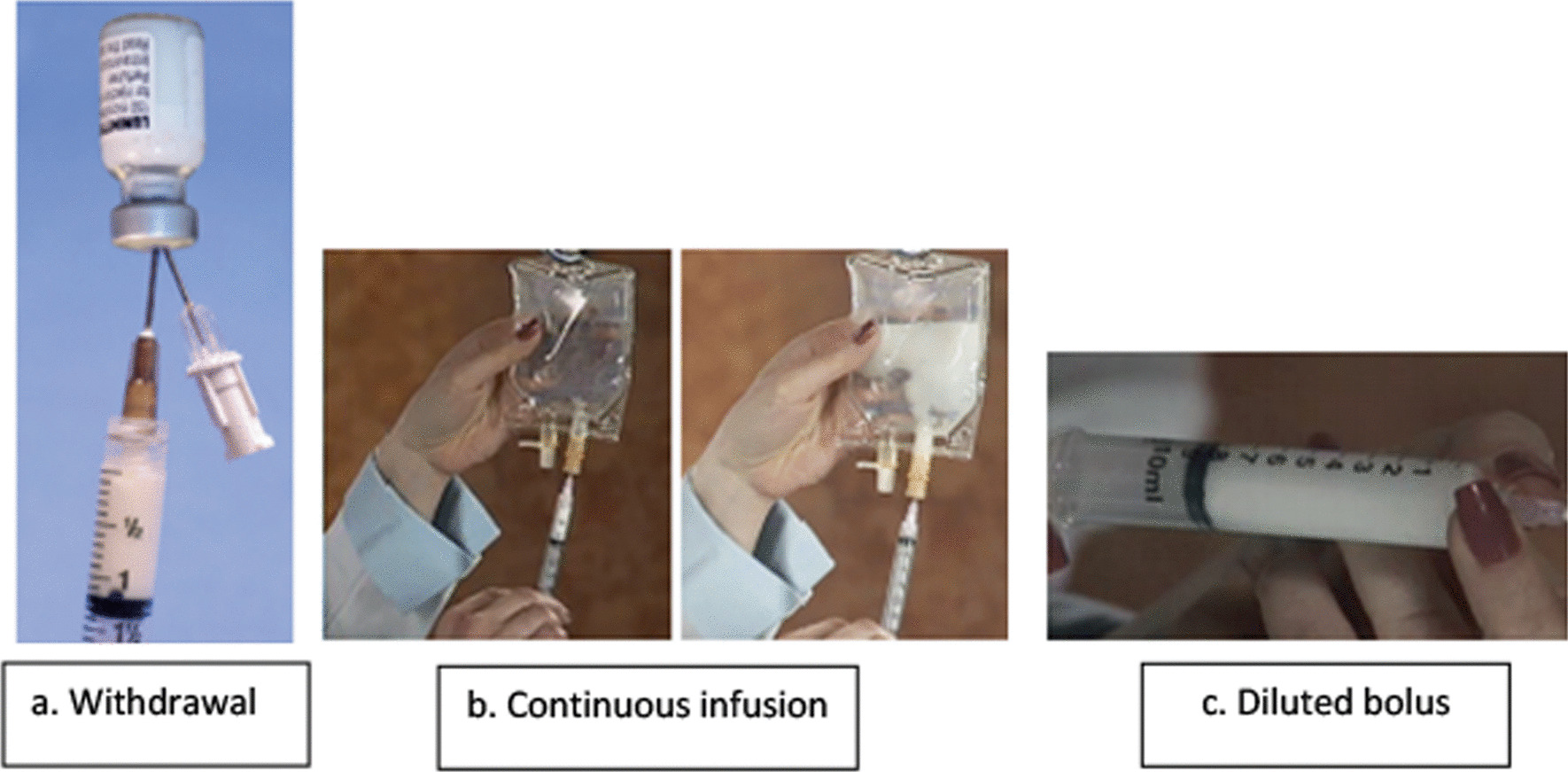
Illustration of the withdrawal of Luminity from the vial using a venting needle, the preparation and administration of a continuous infusion and a diluted bolus injection (Permission to publish images granted by Pharmanovia)

The vial is activated using the Vialmix. Immediately after activation, Luminity appears as a milky white dispersion. If the product stands for more than 5 min after activation, re-suspend with 10 s of hand agitation prior to syringe withdrawal from the vial. The vial should be vented with a sterile syringe needle prior to withdrawing the dispersion: venting avoids negative pressure, allowing easy withdrawal (Fig. 2a). The dispersion should be withdrawn from the vial using a syringe with an 18–20-gauge sterile needle (without a filter). Position the needle to withdraw the material from the middle of the liquid in the inverted vial. No air should be injected into the vial. The product should be used immediately after its withdrawal from the vial. Luminity may be diluted with saline for injection or 50 mg/ml (5%) glucose solution for injection. The contents of the vial are intended for single use only [9].

#### Continuous infusion

Combine 1.3 ml LUMINITY® with 50 ml of saline in an IV bag and squeeze the IV bag gently to distribute the microbubbles evenly (Fig. 2b). Initiate rate of infusion at 4.0 ml/minute, then titrate as necessary to achieve optimal image enhancement. Do not exceed 10 ml/minute [9].

#### Diluted IV Bolus injection

Withdraw 0.2 ml of Luminity with a 1 ml syringe diluted with 0.8 ml saline (Fig. 2c) and administer slowly. The total dose should not exceed 1.6 ml [5].

### Optison

#### Storage

The unopened vial has a shelf life of two years; the finished product after seal perforation has a shelf life of 30 min. Optison should be stored upright in a refrigerator between 2 and 8°C. It is possible to store at room temperature (up to 25 °C) for one day [10].

#### Preparation

Once the seal has been penetrated, the contents should be used within 30 min and any unused product discarded. In the non-resuspended form, Optison has a white layer of microspheres on top of the liquid phase that requires resuspension before use; resuspension is achieved by inverting and gently rotating for approximately 3 min. Complete resuspension is indicated by a uniformly opaque white suspension and absence of any material on stopper and vial surfaces. Do not inject cold solutions taken directly from the refrigerator, allow the vial to reach room temperature and inspect the liquid phase for particulate matter or precipitates before resuspension [10].

Optison should be withdrawn into a syringe within 1 min after resuspension. Avoid pressure instability within the vial by venting the vial with a sterile 18-gauge needle before withdrawing the suspension into the injection syringe. Pressure instability may cause disruption of microspheres and loss of contrast effect. Do not inject air into the vial as this will damage the product [10].

#### Administration

Use the suspension within 30 min after withdrawal. Optison will segregate in an undisturbed syringe and must be resuspended before use. This can be achieved by holding the syringe horizontally between the palms of the hands and rolling it quickly back and forth for at least 10 s. Inject 0.3–0.5 ml as a slow bolus followed by 5 ml of normal saline administered over 10 s.

## Medicines legislation for contrast administration

### Key points

1. There are several pathways that enable different healthcare professionals to administer UCA.
2. All medical professionals can administer UCA, but not necessarily prescribe.

Healthcare professionals who may be required to administer UCA in a contemporary echocardiography department include doctors, cardiac nurses, cardiac physiologists, clinical cardiac scientists, and echocardiographers. There are various routes through which non-medical healthcare professionals can administer UCA (Table 2).

**Table 2:** Non-medical healthcare professionals; prescription and administration matrix

Profession	Supply and administration			Prescribing		
	PSD	PGD	Exemption	SP	IP	IP—CDs
Cardiac physiologist	/					
Clinical scientist	/					
Nurse	/	/	/	/	/	/
Echocardiographer	/					

*PSD* patient specific direction, *PGD* patient group direction, *SP* supplementary prescribing, *IP* independent prescribing, *CD* controlled drugs

The legislation on medicines regulation is defined under the Human Medicines Regulations 2012 (UK statutory instrument) and states that prescription- only medicine can only be administered in accordance with a signed prescription through a Patient Specific Direction (PSD) or Patient Group Direction (PGD) [11].

PGDs and PSDs are created for use in local NHS Trusts, usually in collaboration with a multi-disciplinary group including a doctor, a pharmacist and a representative of any professional group expected to be administering the medicines (e.g., an echocardiographer). It is good practice to involve local drug and therapeutic committees, prescribing committees, and similar advisory bodies.

### Patient group direction

The use of PGDs is written into legislation and provides a legal framework that allows specified healthcare professionals registered with a statutory body overseen by the Health and Care Professions Council (HCPC) to supply and administer specified medicines to pre-defined groups of patients without a prescription [11].

In December 2020, the HCPC published their response to NHS England’s consultation on the proposal for the supply and administration of medicines using PGDs for clinical scientists across the UK. The HCPC supports making amendments to legislation to enable clinical scientists to supply and administer medicines using PGDs [12]. However, at the time of writing this has not been mandated and clinical scientists can only supply and administer medicines using a patient specific direction (PSDs).

### Patient specific direction

A PSD is the traditional written instruction (prescription), signed by a doctor, dentist, or non-medical prescriber for medicines to be supplied and/or administered to a named patient after the prescriber has assessed the patient on an individual basis [13]. By law, anyone whom a prescriber has assessed as competent, and has been delegated the task may follow a PSD [14].

### PSD versus PGD

A PGD can only be used in echocardiography labs where nurses administer UCA. It is therefore recommended introducing a PSD for the administration of UCA, this would cover all healthcare professionals involved in UCA administration.

### Guidance for the administration of UCA in accordance with a PSD

Non-medical clinical staff who administer medicines should receive appropriate and specific training that demonstrates competence in the appropriate procedures. Guidance is given here but individuals should ensure that they also comply with all local requirements. This document aims to aid Trusts in preparing local Standard Operating Procedures, training programmes and assessment of competency for the administration of UCA by non-medical healthcare professionals in accordance with a PSD.

#### Information required in a PSD

**Fig. 3:**
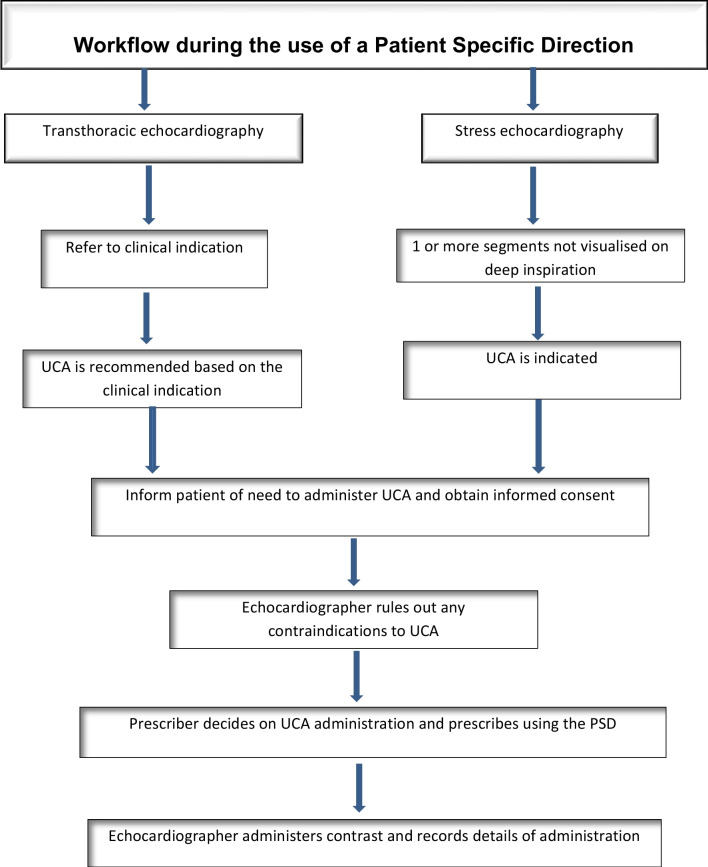
The workflow in an echocardiography laboratory using a PSD

In practice, a PSD is commonly referred to as a prescription by those who write and follow them as this indicates it has been written by a prescriber. Figure 3 demonstrates how the use of a PSD can be incorporated into routine workflow. For the administration of a prescription only medication, a PSD should include the following information as a minimum [15]. See Appendix D for an example:

- Name of patient and other individual patient identifiers
- Allergies or previous adverse drug reactions
- Individual batch number and expiry date of medicine
- Name of medicine and strength
- Route of administration
- Dose
- Frequency
- Date of treatment/number of doses/frequencies
- Signature of prescriber and date PSD written

## Contrast-specific ultrasound imaging

### Key points

1. There are two contrast imaging pre-sets available on the Philips and GE platforms: namely ‘LVO/LV Contrast’ (MI > 0.2) and ‘Low MI/Contrast Low MI’ (< 0.2).
2. The BSE recommends using ‘Low MI’ contrast specific imaging pre-set in the fundamental mode both for rest and stress imaging, if available and well optimised.
3. ‘Low MI’ in the fundamental mode provides excellent endocardial definition, allows low volumes of contrast usages, provides uniform opacification with minimum optimisation and the simultaneous ability to assess perfusion.
4. The ‘LVO’ pre-set in the harmonic mode may be used (ideally fundamental mode) if ‘Low MI’ fundamental imaging mode is not available. LVO imaging mode does not allow perfusion assessment with the presently available UCA, requires a larger volume of contrast and apical artifacts are common.

#### Microbubble physics

Contrast- specific imaging techniques aim to detect the signals from the microbubble (mostly nonlinear signals) and suppress the signals from the tissue (mostly linear signals) by relying on the unique nonlinear behaviour of a microbubble in an acoustic field. Microbubbles in a beam of ultrasound undergo oscillation in response to the variations in acoustic pressure transmitted by the transducer. While oscillating, the microbubbles undergo limited compression but expand more, resulting in non-linear signals which give off both fundamental signals and multiples of incident frequencies called harmonic signals [16]. Tissue generates both fundamental and harmonic signals. The difference between the signals of microbubbles and tissue are the presence of nonlinear signals from the microbubbles. The linear signals from the tissue can be supressed by cancellation algorithms known as contrast specific techniques [17].

Mechanical index (MI) is a measure of acoustic power and is expressed as peak negative pressure/√frequency (*P*- /√ *ƒ*). The MI provides information about the magnitude of energy administered to a patient during an echocardiographic examination. Simply put, the MI is a measure of the power of an ultrasound beam [18]. In this document and subsequent vendor specific recommendations, the following definitions of acoustic power are used; high MI exceeds 0.5, intermediate MI represents values of 0.2- 0.5, low MI represents values < 0.2. Typically, high-MI (> 1) techniques used in conventional harmonic imaging will lead to exponential expansion of the microbubbles, resulting in destruction and elimination of most microbubbles.

#### Contrast specific imaging techniques

Multiple pulses are transmitted down each scan line of the image; pulses are either of alternating polarity or varying amplitude. Returning signals are processed as being derived from tissue, hence suppressed if the returning scatter is perfectly out of phase or proportionally altered in amplitude. The nonlinear signals that remain are derived from contrast microbubbles and are displayed [17].

High and intermediate MI imaging typically employs harmonic imaging techniques (harmonics are more prominent than fundamental signals) and multi-pulse techniques, where low MI techniques use more sophisticated multi-pulse nonlinear detection techniques in the fundamental mode. Low MI techniques rely on fundamental frequencies as harmonic frequencies are very weak at this MI and at the far field causing attenuation. In fundamental mode at the low MI the amplitude of the microbubbles is high, and together with tissue signals which are cancelled through sophisticated multi-pulse cancellation techniques, significantly improves the signal to noise ratio of the microbubbles [19]. There are several cancellation sequences, but the most frequently used is power modulation.

#### Power modulation

**Fig. 4:**
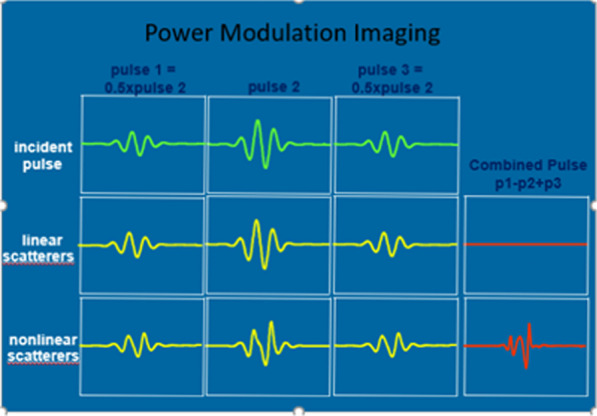
There are multiple consecutive pulses in every scanline, 3 of them are displayed in the top row. Second row represents the corresponding received pulses. The received tissue (linear scatterer) signals are combined by adding the two half amplitude signals and subtracting the full amplitude signal. For tissue, the echoes cancel one another, leading to tissue suppression. The third row represents corresponding signals received from microbubbles (nonlinear scatterers). The shape of the full height pulse is different from the half height pulse resulting in a significant “nonlinear” signal remaining after the sum of the received signals from the two half height pulses are subtracted from the full height pulse (Permission to publish images granted by Philips Healthcare)

The power (amplitude) of each pulse is varied (Fig. 4). The low and high amplitude pulses create a linear response from tissue but nonlinear response from microbubbles. The linear responses from the two different pulses are then subtracted from each other and the transducer only detects the nonlinear behaviour, which emanate only from the microbubbles [1].

The two leading ultrasound suppliers in the UK use distinct contrast imaging techniques on their echocardiography systems, and optimal images are achieved in slightly different ways. A summary of these techniques and their applications across the Philips and GE platforms can be found in Appendix A. Although Philips and GE are the two main platforms being used in the UK with proven capability for UCA imaging, there may be other platforms (Cannon, Siemens, Fuji) with capability for UCA imaging; centres wanting to explore UCA imaging on these platforms should contact the application specialists from the respected vendors.

Table 3 demonstrates the results of selecting default settings for the Philips EPIQ or the GE Vivid E95 system. For standard left ventricular opacification (LVO) the default settings should be sufficient for good quality images, no manual adjustments should be necessary. An optimal LVO image is one in which the LV is uniformly well opacified from the apex to the mitral valve level. There should be clear endocardial border delineation, and the epicardial border should be seen throughout the cardiac cycle. This allows an assessment of wall thickening, the reduction of which suggests pathology.

**Table 3:** Examples of images obtained by utilising default settings on Philips EPIQ or GE Vivid systems

*System* Philips EPIQ CVX system with X5-1 3D probe *Contrast mode* ‘Contrast LVO’ (CGen) pre-set defaults to harmonic mode and an intermediate MI > 0.2 *Focal point* Set at mitral valve level	In this example the default MI is intermediate at 0.30, and the imaging mode is Harmonic. Opacification is not homogenous and there is apical swirling	
*System* Philips EPIQ CVX system with X5-1 3D probe *Contrast mode* ‘Contrast LVO’ (CPen) manually adjusting CGen to CPen changes imaging mode from harmonic to fundamental and MI reduces simultaneously to a low MI < 0.20 *Focal point* Set at mitral valve level	This example was achieved by changing the imaging mode from harmonic to fundamental, which automatically reduces the MI to low (0.19). This results in less bubble destruction and homogenous LV opacification without apical swirling. At this MI there is obvious contrast in the myocardium, but both epicardial and endocardial borders remain discernible	
*System* Philips EPIQ CVX system with X5-1 3D probe *Contrast mode* ‘Contrast Low MI’ (CPen) pre-set defaults to fundamental imaging; low MI < 0.20 with additional tissue cancellation techniques operating in the background *Focal point* Set at mitral valve level	The ‘low-MI’ pre-set has several algorithms which optimises imaging. Despite superficially similar settings to the previous example, here the signal-to-noise ratio is improved such that microbubbles can be seen in the myocardium at a higher intensity. This makes both endocardial and epicardial borders clearer, no bubble destruction is seen in the nearfield, and opacification is homogeneous However, the presence of myocardial apical defect can be overcome moving the focal point towards the apex as explained below	
*System* GE Vivid E95 system with a M5Sc 2D probe *Contrast mode* ‘LV Contrast’ pre-set (harmonic imaging; intermediate MI at 0.21) *Focal point* Set at mitral valve level	In this example, apical swirling may result, this mode of imaging relies on an intermediate MI leading to some bubble destruction in the nearfield	
*System* GE Vivid E95 system with a M5Sc 2D probe *Contrast mode* ‘LVO Stress’ pre-set (harmonic imaging; intermediate MI at 0.21) *Focal point* Set at mitral valve level	The images vary slightly from the LV Contrast images since the LVO Stress mode has been designed for acquisition at higher heart rates and continuous capture	
*System* GE Vivid E95 system with a M5Sc 2D probe *Contrast mode* ‘Contrast Low MI’ pre-set (harmonic imaging; low MI at 0.10) *Focal point* Set at mitral valve level	Image resolution is not optimal, myocardial opacification is likely to be background noise and not true perfusion. However, GE is currently refining this mode of imaging	

If imaging for perfusion, which can only be performed at low MI for the commercially available microbubbles, some minor adjustments will need to be made to optimise images. It is recommended that these adjustments are made with the help of your vendor-specific imaging specialist and a separate pre-set be created for perfusion imaging. There is no one specific setting that can be recommended to fit all the different systems; they will vary slighty between the different software versions and also between 2 and 3D probes. For the assesment of myocardial perfusion, the myocardium should be well opacified throughout. At rest, when the wall thickening is normal, there should not be any reduction in myocardial opcification. For both settings, the focal point should be set at the mitral valve level. A working example of the settings for an optimal ‘Low MI’ pre-set (Philips EPIQ) can be seen in Appendix B. Settings are similar between 2 and 3D probes, however, the 2D probe is recommended for myocardial perfusion imaging with a low MI pre-set.

## Routine clinical applications

### Key points

1. The BSE recommends that contrast is used when two or more segments cannot be visualised.
2. Contrast may be considered for detailed assessment of LV ejection fraction and LV volumes when accurate assessment is central to clinical decision-making.
3. If LV ejection fraction is being monitored, contrast should be considered even if all segments are seen.
4. Contrast may be considered in the assessment of intracardiac masses, apical abnormalities, and non-compaction.

### Assessment of cardiac function and structure.

#### LV volumes

**Fig. 5:**
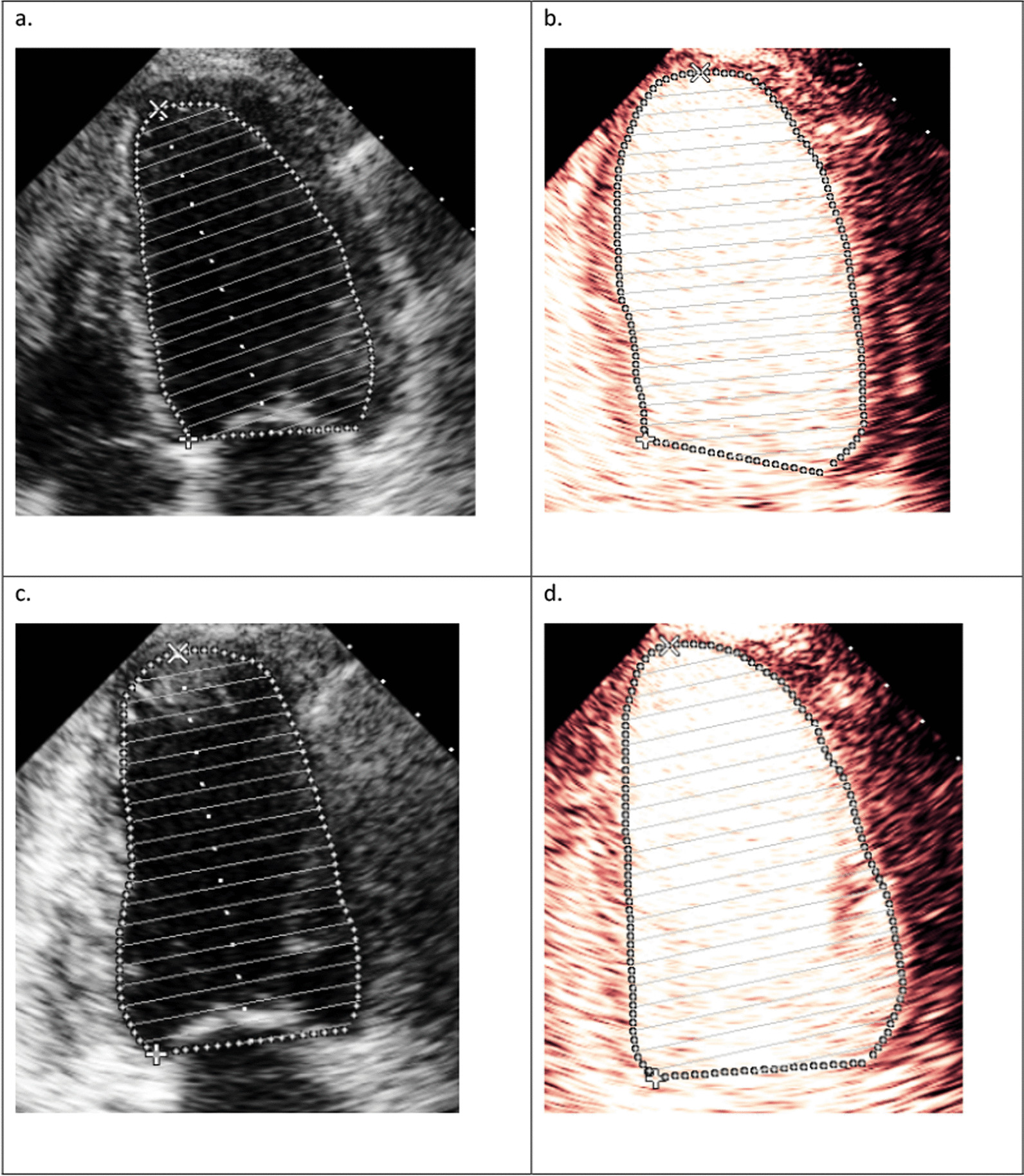
**a** Unenhanced apical 4 chamber with a diastolic LV volume of 103 ml. **b** Contrast enhanced apical 4 chamber in diastole measuring a LV volume of 114 ml. **c** Unenhanced apical 2 chamber measuring a diastolic volume of 105 ml. **d** Contrast enhanced apical 2 chamber in diastole measuring a LV volume of 127 ml

Volumetric measurements should be made by tracing the interface of the compacted myocardium and the LV cavity, excluding the trabeculae, as seen in Fig. 5b and d, below [20]. This may not be reliably performed with unenhanced imaging as trabeculation can obscure detection of compacted myocardium. When there is poor endocardial visualisation of at least 2 contiguous segments out of 6 segments in the apical views, UCA is recommended. Unenhanced 2D images may underestimate LV volume assessment because of noise in the LV cavity and foreshortening. Contrast echocardiography not only suppresses noise from the LV cavity, improving identification of the compacted myocardium, but also minimises foreshortening which allows the true longitudinal axis of the LV to be measured [2]. Irrespective of whether 2D or 3D imaging is being utilised, LV volume measurements obtained with UCA are significantly larger than unenhanced images (Fig. 5) [21], and are closer to values achieved with MRI, considered a gold standard for LV volume assessment. The normal range of LV volumes with UCA has not been established, therefore use chamber quantification guidelines with caution when applying normal ranges.

#### Left ventricular ejection fraction

Even when image quality is good, UCA may be considered in patients where accurate quantitative assessment of left ventricular ejection fraction (LVEF) is central to clinical decision-making. Such scenarios may include consideration for device therapy, monitoring the effects of potentially cardiotoxic medication (e.g., chemotherapeutic agents), or in patients with valve disease who are under surveillance for intervention [2, 3]. UCA increases the accuracy of LVEF measurements and reduces inter-observer variability [21]. During routine transthoracic echo (TTE) studies, UCA should be considered when two or more out of 6 LV segments in the apical views cannot be visualised adequately for the assessment of LV function (LVEF or regional assessment) [2, 3, 16]. Once a decision has been made to employ UCA, it is imperative that subsequent surveillance studies use the same technique for comparison and identification of any interval change.

#### Regional wall thickening

Where views can be obtained but endocardial border delineation is insufficient, UCA should be considered. The assessment of wall thickening is highly subjective and highly dependent on image quality, emphasising the importance of being able to detect both the endocardium and epicardium throughout ventricular systole [2, 3]. Analysis of regional wall thickening is subject to significant inter-observer variability; therefore, agreement is improved with the use of UCA [22].

### Cardiac structure

Besides visualisation of the endocardial borders, opacification of the LV cavity with UCA allows for the detection, characterisation, and diagnosis of intracardiac abnormalities and conditions that influence the shape and contour of the LV [2–4].

#### LV apical hypertrophy

When apical hypertrophic cardiomyopathy is suspected but cannot clearly be excluded with routine unenhanced echocardiography, UCA should be administered. Visualisation of apical aneurysm and thrombi associated with apical hypertrophy can also be improved.

#### LV non-compaction

LV non-compaction is alterations of myocardial structure with thickened, hypokinetic segments consisting of two layers: a thin, compacted subepicardial myocardium and a thicker, noncompacted subendocardial myocardium. UCA is useful in identifying the deep recesses and exposing the myocardial trabeculations in the non-compacted myocardium [1–3]. Low MI imaging may be used but if not diagnostic reassess using an MI setting that is higher (LVO with Intermediate MI) with harmonic setting as cardiac structures reflect harmonics.

#### LV apical thrombus

A thrombus will appear as a ‘filling defect.’ UCA increases the sensitivity for the detection of a LV thrombus and improves the negative predictive value [24]. Echocardiography is the initial tool for identification of apical thrombi [25]. The BSE recommends that UCA is considered in all patients with either (i) an apical aneurysm, or (ii) akinesis of all four apical segments (including the apical cap), irrespective of image quality. It is important to appreciate that even when UCA is employed in this clinical scenario, the negative predictive value of contrast echo for apical thrombus is not 100%, and additional imaging may be required [26]. In the absence of an apical aneurysm and/or apical akinesis, where there is suspicion of a mass, UCA should be administered if image quality is suboptimal.

Non-traditional “off-axis” views should be obtained to visualize the entire apex while imaging with UCA. LVO imaging mode may also be used but often small thrombi detection may be compromised by overlying apical swirling due to microbubble destruction at higher MI.

#### LV apical aneurysm

True aneurysms are characterised by thin walls and a dilated apex, which involve the full thickness of the ventricular wall and may be akinetic or dyskinetic. If the apex is not visualised adequately an apical aneurysm may go undetected until UCA is administered. A false aneurysm occurs when the LV ruptures after acute myocardial infarction but is contained by fibrinous material. This is frequently a terminal diagnosis although on occasion life-saving surgery can be undertaken. UCA may provide value in identifying false aneurysm.

#### Left atrial appendage thrombus

In patients with atrial fibrillation planned for cardioversion, whenever the atrial appendage has significant spontaneous contrast or cannot be adequately visualized during unenhanced transoesophageal echocardiography (TOE), UCA should be considered. Specific MI settings have not been established, however, for optimal imaging switch to harmonic mode at a transmit frequency of 3 MHz (transmit with the MI reduced to < 0.2 in the power modulation mode if available), with focus set at the level of the appendage.

#### Intracardiac masses

**Fig. 6:**
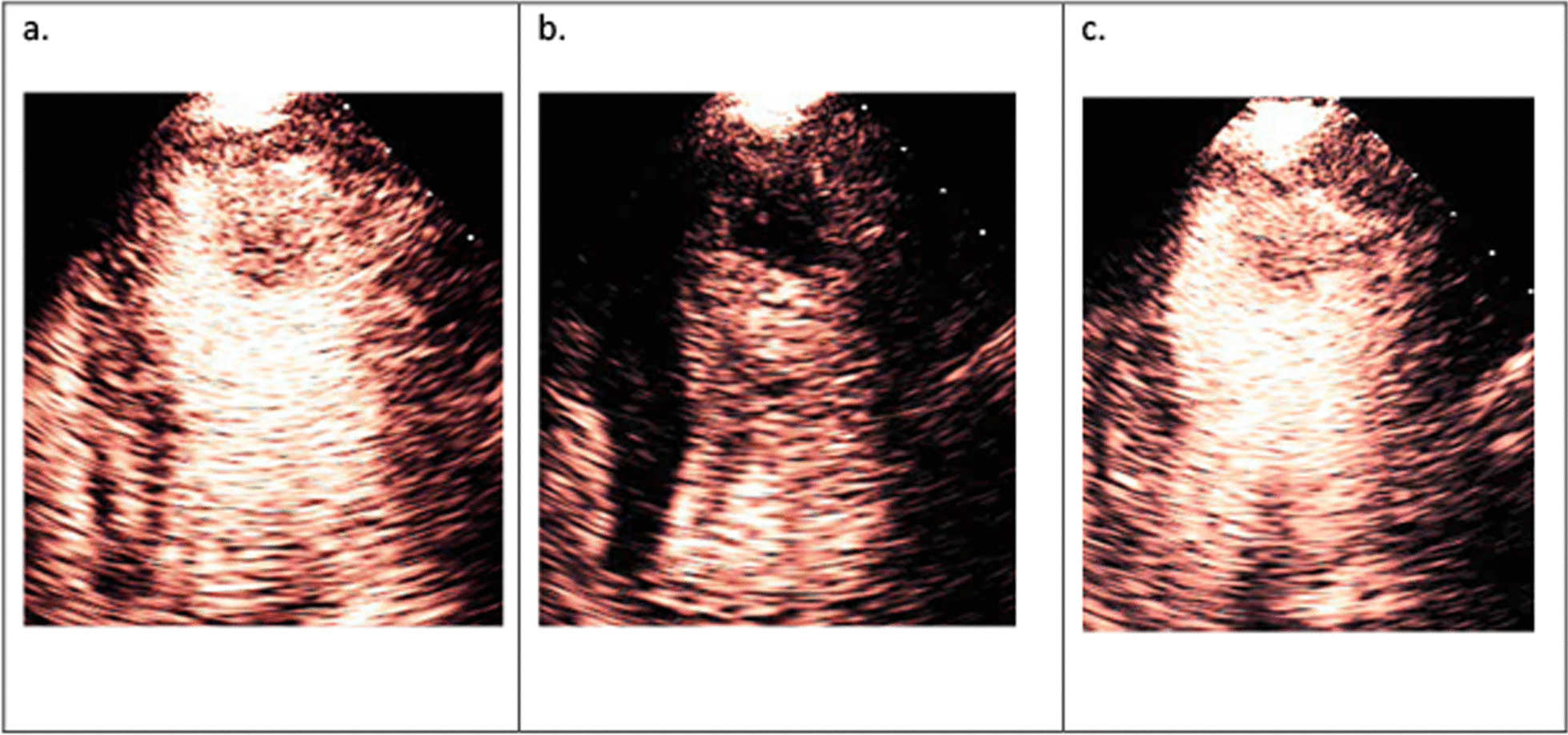
**a** Steady state perfusion reveals a mass in the LV apex perfused with microbubbles. **b** Microbubbles are transiently destroyed with a high MI flash. **c** There is replenishment of microbubbles seen in the mass post flash suggesting a highly vascular structure, this is highly suspicious of a tumour

Intracardiac masses can be pathological such as thrombus, vegetation, or tumour; or normal variations of cardiac structure such as a false chord, accessory papillary muscle, or prominent trabeculation. Any suspicious cardiac mass, when not clear on baseline images, may be confirmed or refuted after administration on UCA, once structures are clearly delineated [27]. Perfusion imaging, described later, helps in characterising the mass. Vascular masses (exhibiting presence of prominent perfusion) are often associated with malignant tumours (Fig. 6). Myxomas demonstrate patchy perfusion. An avascular mass (demonstrating no contrast uptake) overlying an akinetic myocardium is highly likely to be a thrombus. Off-axis images and longer loop acquisitions are recommended to help identify and characterize intracardiac thrombi or masses [23].

### Right ventricular structure and function

When trying to diagnose regional wall thickening abnormalities, tumours, and thrombi in the right ventricle (RV), UCA administration can be used to distinguish these abnormalities from normal structures. To position the RV in the near field, use the modified apical four-chamber window of the RV (RV focussed view) or the parasternal view [2]. Functional assessment of RV can also be achieved using UCA [28]. However, wall thickening abnormalities can be difficult to detect due to the thin right ventricular free wall and lack of appreciation of the epicardial border.

## Stress imaging

### Key points

1. The threshold for using UCA during stress echocardiography should be extremely low for optimal assessment of regional wall motion abnormalities.
2. When myocardial perfusion assessment is required, UCA should be administered even if all segments are visualised.
3. Where expertise exists, myocardial perfusion could be assessed in patients undergoing dobutamine, bicycle or vasodilator stress and following treadmill if likelihood of CAD is high, for the assessment of myocardial ischaemia and viability beyond wall motion assessment.

UCA use during both exercise stress echocardiography (ESE) and dobutamine stress echocardiography (DSE) improves sensitivity, specificity, and accuracy in patients with suboptimal images, owing to better endocardial border detection at rest and during stress [2, 3, 16]. The use of UCA when two or more segments are not adequately visualized, either at rest or during peak stress, reduces costs and improves cost saving with abnormal results predicting adverse events [2, 29]. Because disease in a single coronary territory may affect only one segment in any apical or parasternal view, the BSE proposes that UCA be considered if any segment cannot be adequately visualised at rest. UCA should also be considered if deep inspiration at rest (which mimics breathing during exercise stress) leads to poor visualisation of one or more segments.

### Imaging for myocardial perfusion

#### Pathophysiologic basis of UCA- assessed myocardial perfusion

**Fig. 7:**
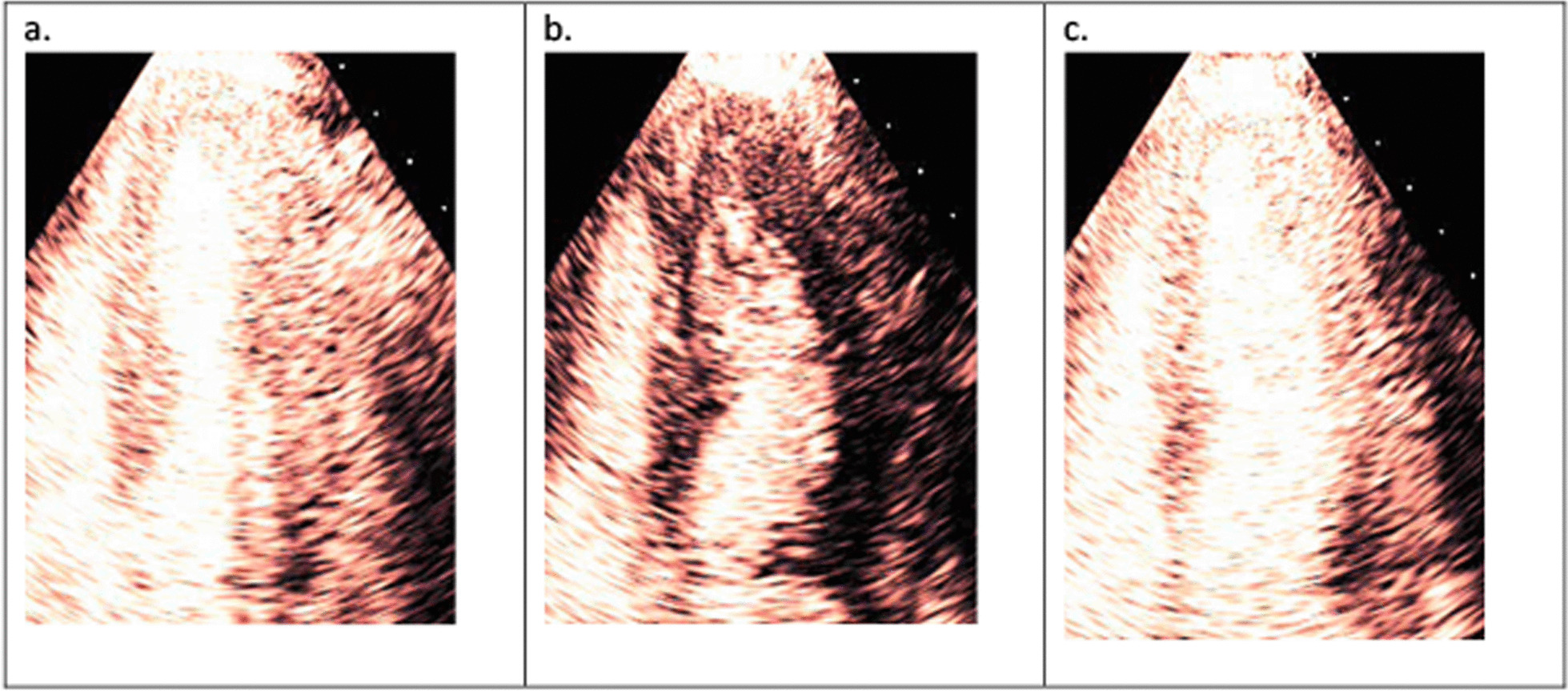
**A** At peak stress a steady state of contrast is seen throughout the myocardium. **B** A high MI flash is delivered, and microbubbles are temporarily cleared from the myocardial capillaries. **C** Myocardial replenishment of microbubbles are seen after one cardiac cycle, with an artefact in the basal anterolateral segment (see Additional file 1: Video S1). Artefact is commonly seen here as lateral resolution is lower than axial definition. The artefact is characterised by full thickness defect which extends beyond the myocardium as opposed to a true defect which is usually subendocardial and fills from the epicardium to the endocardium after 2 secs (see text)

Myocardial perfusion is tissue blood flow at the capillary level, 90% of blood within the myocardium resides within the capillaries [3, 30]. Using a continuous infusion of UCA will result in a steady state within the circulation (Fig. 7a), at which point the signal intensity in the myocardium depicts capillary blood volume [31].

The best imaging mode to assess myocardial perfusion using the commercially available UCAs is as for assessing cardiac structure and function -low MI contrast specific imaging. Microbubbles are temporarily cleared from myocardial capillaries by transiently increasing the MI to a maximum of 0.9MI (generally 0.6–0.9) (Fig. 7b), following which low MI imaging is commenced. Microbubbles will replenish the myocardium (Fig. 7c). The rate of replenishment determines myocardial capillary blood velocity, and the product of capillary blood volume x velocity denotes myocardial capillary blood flow (MBF) [3].

#### Qualitative assessment of CAD

**Fig. 8:**
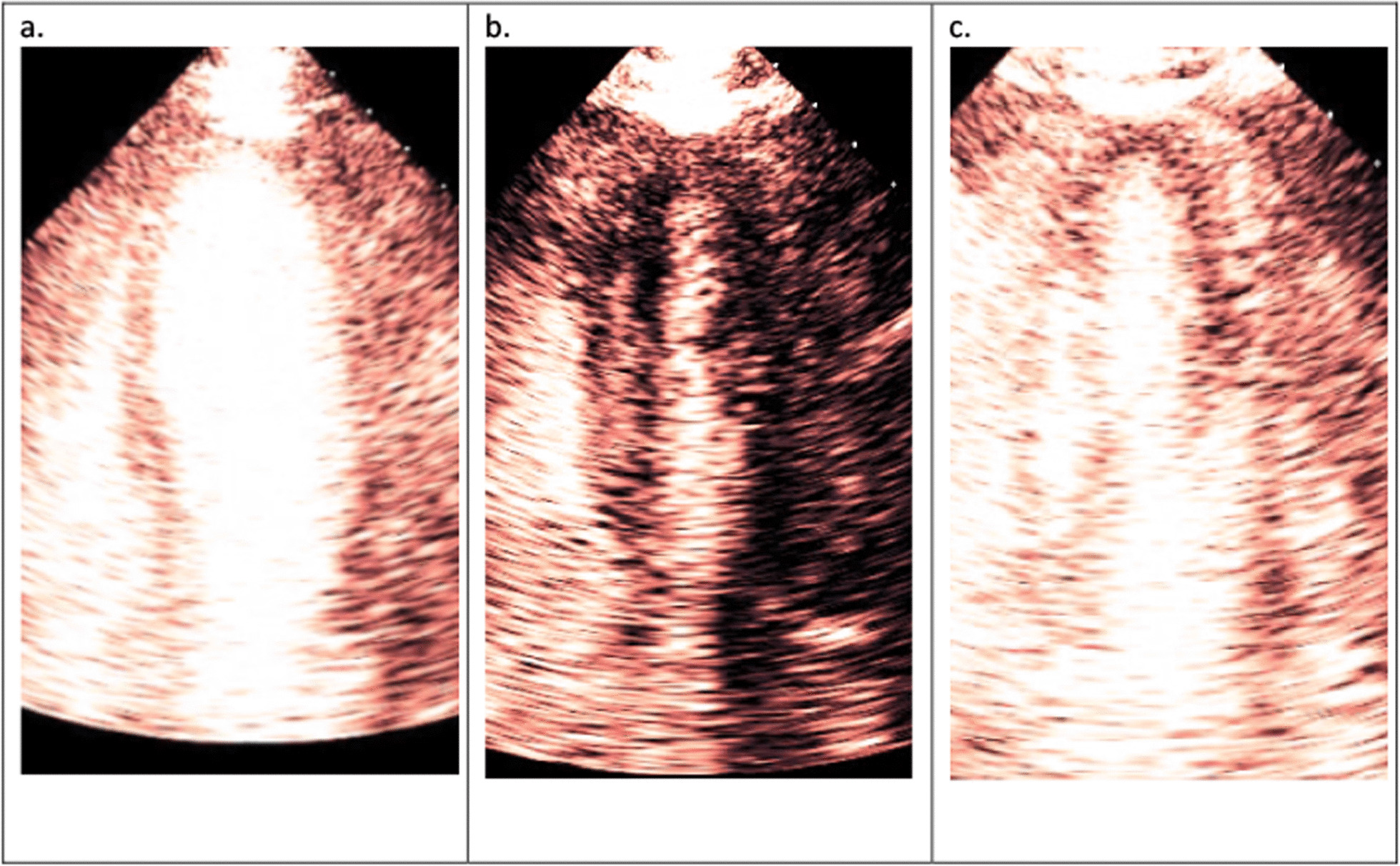
**a** Steady state of myocardial perfusion at peak stress with a heart rate of 120 beats per minute. **b** Microbubbles are cleared with a high MI flash. **c** Delayed appearance of contrast with a reduced intensity in the sub endocardium seen in the apical segment after 4 cardiac cycles (see Additional file 2: Video S2)

In patients with flow-limiting CAD, both capillary blood velocity and capillary blood volume are reduced in proportion to the severity of coronary stenosis. At rest, a healthy myocardium supplied by a normal coronary artery will replenish within 5 s following a series of high MI frames (five cardiac cycles if the heart rate is 60 bpm) because normal capillary blood flow velocity is 1 mm/s, and the elevation of the transducer is 5 mm. During stress, MBF normally increases four–fivefold, such that replenishment will be complete within 1–2 s (2–4 cardiac cycles at a heart rate of 120 bpm). Typically, the delayed appearance of contrast with a reduced intensity in the sub endocardium (Fig. 8), is the hallmark of flow-limiting CAD [31]. However, myocardial ischemia can also occur in patients without obstructive CAD due to coronary microvascular dysfunction (CMD). A condition more common than previously thought in patients presenting with ischemic symptoms, known as Ischemia in Non-Obstructive Coronary Artery Disease (INOCA). In this condition regional wall thickening abnormalities may not occur for two reasons: (1) it is commonly a global phenomenon (2) unlike CAD, the subendocardium may not be affected—a prerequisite for occurrence of wall thickening abnormalities. The characteristic myocardial perfusion abnormality during stress in INOCA is slow filling of the myocardium beyond 2 s. However, capillary derecruitment with resultant perfusion defects may also occur. Quantitative myocardial contrast echocardiography (MCE) assessment of myocardial flow reserve < 2 is diagnostic of INOCA [23, 32, 33].

**Fig. 9:**
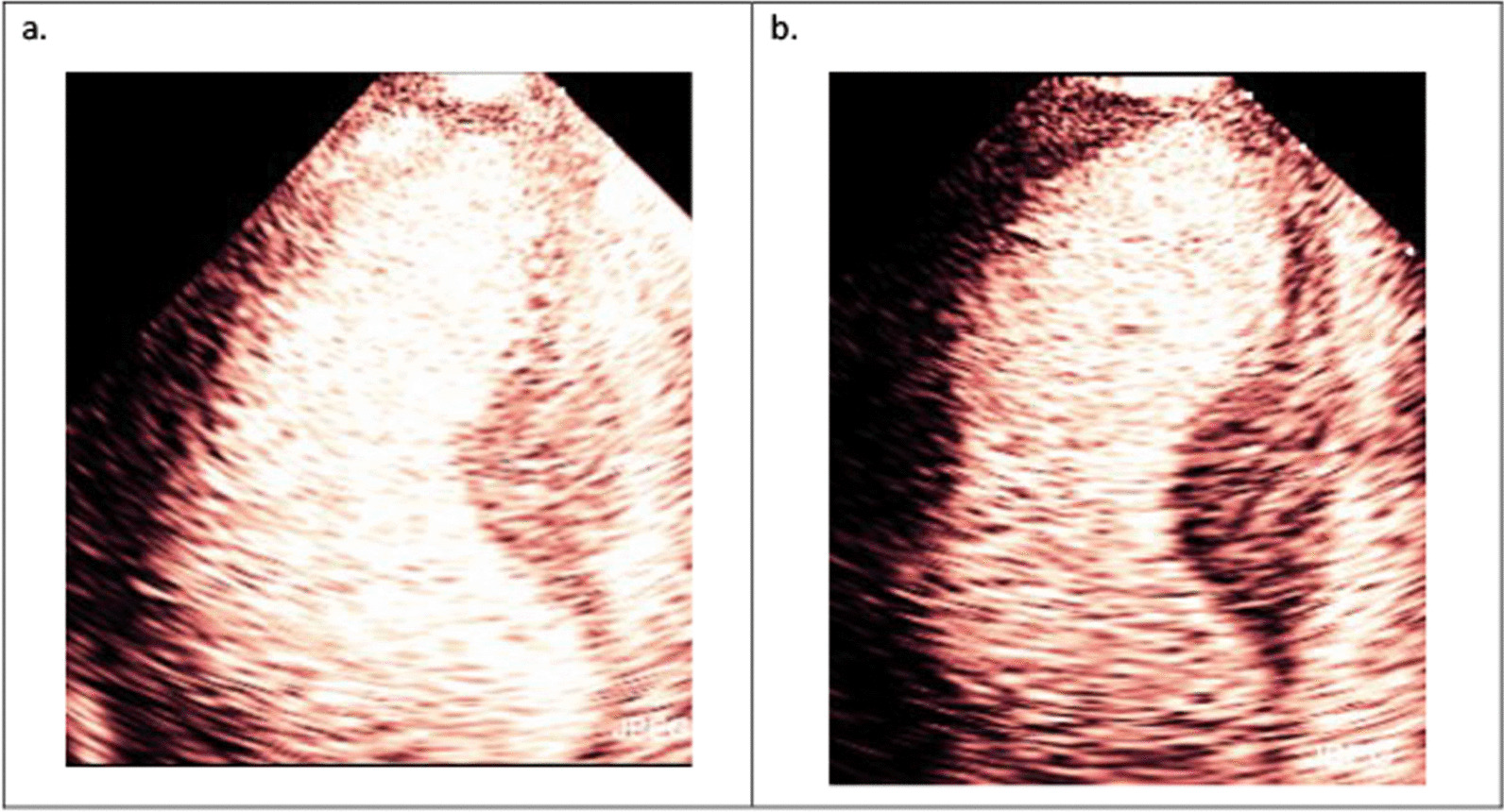
**a** With focussed view of the septum, which is affected during LBBB, wall thickening at end diastole is shown. **b** Wall thickening at end systole is shown. There is no discernible increase in wall thickening suggestive of possible ischaemia in the septum

**Fig. 10:**
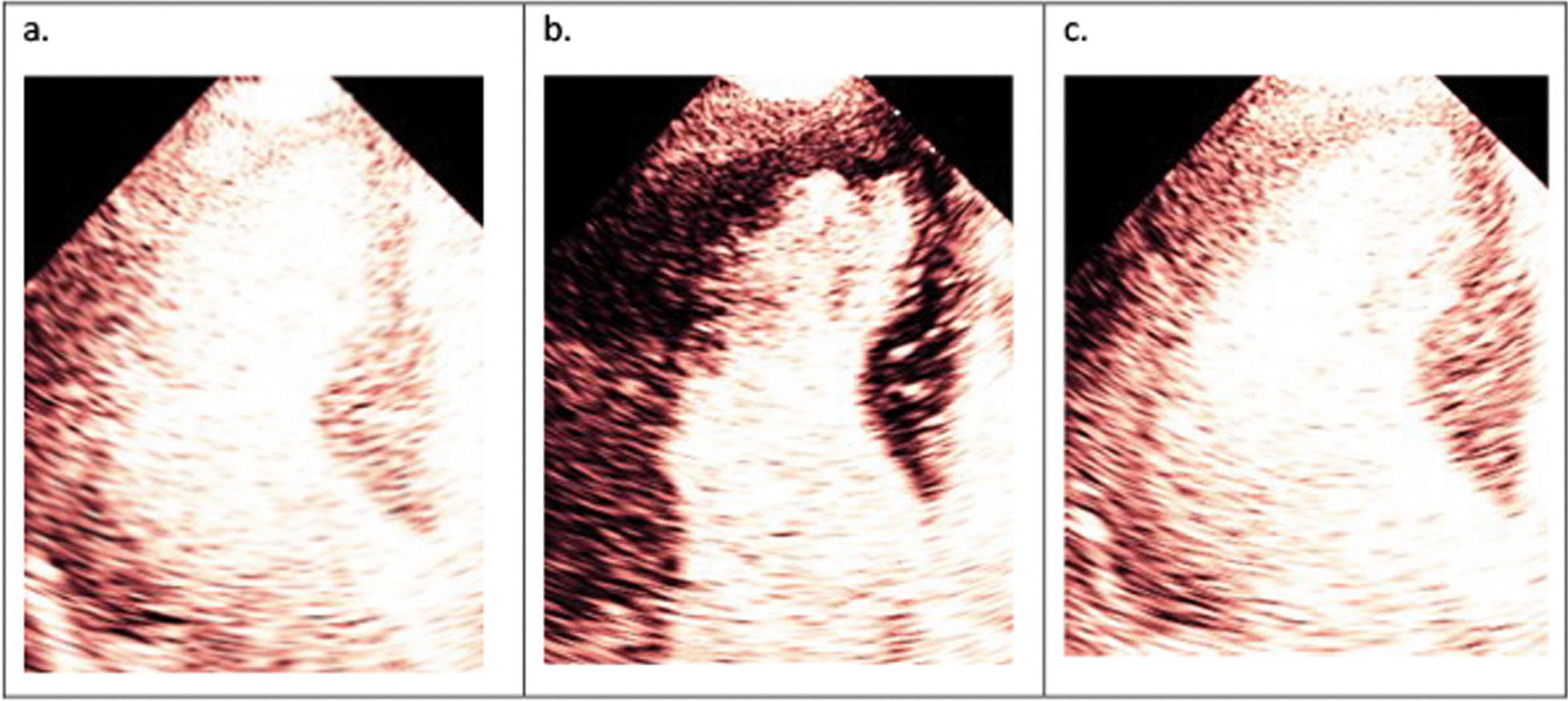
**a** Steady state of myocardial perfusion at rest is shown in the same patient as Fig. 9. **b** Microbubbles are cleared with a high MI flash. **c** Myocardial replenishment of microbubbles are seen after one cardiac cycle, suggesting normal coronary blood flow, hence no myocardial ischaemia despite reduced wall thickening (see Additional file 3: Video S3)

Perfusion provides incremental diagnostic and prognostic information beyond wall motion abnormalities (WMA) during stress echocardiography. Perfusion analysis during demand stress such as exercise and DSE improves the detection of CAD compared with WMA alone [1, 3]. It is also recommended that perfusion assessment be performed in patients with left bundle branch block (LBBB), as it has it has been shown that perfusion remains unaffected despite LBBB inducing WMA which is due to abnormal activation sequence of the myocardium [34] (Fig. 9). Therefore, inducible perfusion defect in this scenario will suggest flow-limiting CAD, whereas an inducible WMA may not [35, 36] (Fig. 10). Vasodilator stress may be performed when demand stress is contraindicated. Since this test does not induce increased oxygen demand, WMA assessment is less sensitive than perfusion imaging for the detection of flow- limiting CAD [30]. Vasodilator stress is preferable in patients with suspected INOCA. Myocardial perfusion assessment may also detect hibernating myocardium because the presence of perfusion in dysfunctional myocardium suggests myocardial viability which is likely to improve following revascularisation [37]. The details of how to perform UCA-enhanced myocardial perfusion for the detection of myocardial ischemia and myocardial viability is detailed in a recent European Association of Cardiovascular imaging (EACVI) publication [38]. Appendix C provides practical guidance on how to perform and interpret a myocardial perfusion study.

## Non-routine application of contrast imaging

### Key points

1. In the assessment of aortic disease.
2. In congenital heart disease that presents challenging windows, UCA may improve image quality and help with quantification of ventricular function.
3. In intensive care units where patients are ventilated, and imaging is technically challenging.
4. To enhance Doppler signals during assessment of RVSP.

### Aortic pathology

Although CT and TOE are the most common diagnostic modalities for the detection of type A aortic dissection, contrast enhancement of the aorta can assist in distinguishing a true intimal flap from linear artifact on both TTE and TOE. In patients with aortic dissection, contrast enhancement can also help delineate the true and false lumens. The initial bolus of UCA needs to be imaged during the first pass to delineate the differential flow in the true and false lumens, avoid attenuation from too large/rapid injection [2].

### Contrast imaging in the intensive care unit

Echocardiography is often technically difficult in ITU patients due to mechanical ventilation, wound dressings, and difficulty in patient positioning, emphasizing the need for UCA in this patient population. UCA is recommended in all technically difficult ITU patients to diagnose potentially life-threatening conditions quicker and more accurately and to reduce the need for downstream diagnostic testing. UCA should not be withheld based on any diagnosis or co-morbidity [23].

### Contrast imaging in paediatric echocardiography

Contrast agents have now been licensed for use in paediatric cardiology [46].

### Contrast imaging in congenital heart disease

Patients with CHD pose additional challenges due to acoustic window limitations from previous cardiac operations, chest wall issues, and alterations in cardiac geometry. It has been shown that UCA improve visualization of segmental wall motion in both the left and right ventricles in patients with CHD, leading to better quantification of ventricular function at rest and during physiologic or pharmacologic stress [2].

### Contrast imaging for Doppler enhancement

The peak tricuspid regurgitant (TR) jet velocity measured by continuous wave Doppler is used in the estimation of the right ventricular systolic pressure (RVSP). Trivial TR can result in incomplete Doppler spectral signals and an underestimation of the true pressure gradient. UCA or agitated saline may be used to enhance the TR envelope for more accurate assessment of velocity. Similarly, on the left side of the heart, UCA can help improve the spectral Doppler assessment of the peak aortic jet velocity [39]. When using UCA for Doppler enhancement, spectral gain should be adjusted to minimise artefact and avoid overestimation of velocities. If required, it is important that subsequent surveillance echo studies also employ UCA for comparison and detection of interval change.

When using UCA for Doppler enhancement, the volume/concentration should be significantly less than a standard dose for LVO. Excess UCA will result in high levels of background noise; wait for the contrast to clear and reduce the spectral gain to further reduce background noise, this will enhance the intensity of the Doppler envelope and avoid overestimation of velocities.

## Imaging artefacts

### Key points

1. Common artefacts include apical swirling, basal attenuation, blooming, rib, and lung artefacts.
2. Artefacts are more common with LVO pre-sets.
3. These can be overcome by paying attention to the speed of contrast administration, transmit focus adjustment, moving the transducer and bringing the specific part to the centre (axial) of the imaging plane.

### How to recognise and overcome common artefacts during LVO

Table 4 describes commonly encountered artefacts when using UCA enhanced echocardiography and provides techniques to overcome these limitations.

**Table 4:** Imaging artefacts

Swirling		
*Description* Swirling of contrast, especially in the nearfield, is common, particularly when the LV is dilated and there is impaired function (Additional file 4: Video S4) This results in poor endocardial border definition and poor appreciation of apical pathology like thrombus which may be overlooked This is more often seen in LVO mode as the MI is higher which results in destruction of UCA	*Solution* 1. If available, use ‘Low MI’ imaging, the low MI will significantly minimise bubble destruction 2. Where low MI is not available, change the imaging mode from harmonic to fundamental (CGen to CPen) in the LVO mode. This will automatically reduce the MI but maintain optimal imaging 3. Consider moving focal point towards the apex which will reduce UCA destruction (with a near field focus, the beams in the near field become much narrower and will not overlap, reducing the energy each bubble is exposed to) 4. If swirling is observed throughout the LV, increase the rate and/or the volume/concentration of UCA injection	
Attenuation		
*Description* Results from a high volume/concentration of microbubbles in the apex, causing a near-field backscatter and acoustic shadowing of far-field structures Manifests as a dark shadow in the far-field, particularly at the base of the LV making delineation of the endocardial borders impossible (Additional file 5: Video S5)	*Solution* 1. Lower the dose of UCA (volume/concentration) being injected 2. Slow the rate of bolus injection or of continuous infusion 3. Brief application of high MI imaging or colour doppler will clear the excess microbubbles and rapidly resolve the artefact 4. Remember: an inexperienced echocardiographer might assume more contrast is required to overcome the shadow. However, the exact opposite is required	
Blooming		
*Description* Often follows attenuation. Gives the appearance of contrast within the myocardium and could be mistaken for perfusion (but IS NOT perfusion) With blooming artefact, contrast signals are spread beyond the tissue into neighbouring regions (Additional file 6: Video S6) This results in poor delineation of endocardial borders and small thrombi can also go undetected	*Solution* 1. Reduce the dose of UCA (volume/concentration) and/or slow down the rate of bolus injection 2. A good technique is to stop injecting as soon as a streak of UCA is observed in the RV and if required inject more; giving too much too soon will result in blooming 3. If using a continuous infusion, reduce the rate of infusion 4. If blooming occurs during SE at peak stress, every attempt should be made to promptly destroy the microbubbles by briefly applying high MI imaging or colour doppler to clear excess microbubbles and reverse blooming 5. The key to avoid attenuation/blooming artifact is slow injection	
*Description* A series of events following the injection of a high concentration/volume, with apical blooming followed by basal attenuation and a gradual clearance of UCA allowing for better endocardial border definition at the apex and base (Additional file 7: Video S7)		
Rib / papillary muscle artefacts		
*Description* Rib artefacts are seen in the lateral wall of the apical four-chamber view and result from adjacent ribs obstructing the transmission of ultrasound from the transducer in the lateral scan planes (Additional file 8: Video S8). This can similarly be seen in the apical four- and two-chamber views where the papillary muscles obstruct the transmission of ultrasound in the same way	*Solution* 1. Moving the probe footprint to adjust the image orientation will usually compensate for this artefact	

### How to recognise and overcome common artefacts during perfusion imaging

Misdiagnosis of a perfusion defect, characterised by the absence of contrast, can be made in the presence of shadowing from ribs/lung tissue or the movement of the heart in and out of the scan plane. This can be avoided by adjusting the transducer on the chest or by asking the patient to breath hold during image acquisition. Apical defects can occur because of excessive contrast destruction due to relatively high acoustic power in the near field. This is usually transmural with adjacent LV cavity bubble destruction. This can be overcome by moving the transmit focus to the apex. Basal anterior and anterolateral artifacts are common due to reduced transmit power in the lateral part of the sector. This can be overcome by bringing this part of heart into the axial plane.

## Limitations of contrast echocardiography

It is possible to achieve good quality recordings while using UCA in most patients; however, echocardiographers require training and a good understanding of the physics of microbubbles as well as the relevant imaging techniques. There are specific artefacts unique to contrast echocardiography and echocardiographers should be able to recognise and eliminate them (as discussed earlier). A limitation to the use of UCA in echocardiography may be the additional cost. However, alternative imaging methods such as nuclear imaging or CMR are more expensive, and there is good evidence to suggest suboptimal echocardiographic studies lead to increased downstream costs [3].

## Clinical safety of UCA

### Key points

1. Incidence of severe reactions to UCA is extremely low (1 in 10,000 administrations).
2. Contrast agents are contraindicated in patients with prior hypersensitivity.
3. UCA are NOT contra-indicated in the presence of cardiac shunts or pulmonary hypertension.

### General safety

The safety of UCA has been examined in a wide variety of patient populations that include inpatients, outpatients, critically ill patients, patients with pulmonary hypertension, patients on mechanical circulatory support and patients undergoing stress echocardiography [40, 40–43]. These studies have concluded that life-threatening reactions to UCA are extremely rare and occur in approximately one in 10,000 doses [41]. The most serious adverse events include hypersensitivity reactions that are not thought to be immunoglobulin E-mediated, but rather pseudo-anaphylactic reactions from complement activation [44, 45]. Recently the potential for rare IgE-mediated hypersensitivity reactions to certain components in commercially available lipid-based contrast agents (Luminity, SonoVue) has resulted in an alert on the safety of UCA [46]. Luminity and SonoVue contain Polyethylene Glycol (PEG) in either the excipient alone or in the microbubble shell. Hypersensitivity reactions to PEG components have recently been recognised after the publication of case reports implicating PEG allergy. Health and care providers should enquire about hypersensitivity to agents that contain PEG as their active ingredient, which include bowel preparations used during colonoscopy and certain laxatives. In the case of potential PEG allergy, Optison, which does not contain PEG, should be used.

### Contraindications and specific safety considerations

To date, there are no safety data on pregnant patients. However, recently FDA approved Lumason (SonoVue in Europe) for intravenous use in the paediatric population at a dose of 0.03 ml/kg as bolus [47].

All contraindications for UCA use in patients with known or suspected right-to-left, bi-directional, or transient right-to-left cardiac shunts have been removed (Luminity, SonoVue and Optison) [23]. Similarly, there are no concerns for the use of UCA in patients with pulmonary hypertension, with several retrospective and prospective studies demonstrating the safety of these agents in such populations [39].

The only contraindication to Luminity is known or suspected hypersensitivity to perflutren and or lipid shell. SonoVue is contraindicated in patients with a history of hypersensitivity reactions to sulfur hexafluoride, lipid microsphere components or to any of the inactive ingredients of SonoVue. Optison is contraindicated in patients with known or suspected hypersensitivity to perflutren, blood, blood products, or albumin [2].

### Adverse events

Adverse reactions include shock, throat swelling, facial swelling, hives, seizures, convulsions with the most critical concern during anaphylaxis being respiratory distress due to bronchospasm [3, 23].

Other reported adverse events associated with UCA are rare and mild, include headache, weakness, fatigue, palpitations, nausea, dizziness, dry mouth, altered sense of smell or taste, dyspnoea, hives, and back pain. UCA administration should stop immediately stop if there are any signs of adverse events [3, 23]. Adverse events should be recorded in the patients’ notes and within the echocardiogram/stress echocardiogram report to avoid repeat administration.

Back pain as a side-effect is mostly associated with Luminity. The leading hypothesis is that it is related to a complement mediated idiosyncratic reaction, also observed with other injectable agents that contain lipid membranes. If back pain occurs during Luminity administration, immediately stop injection, and monitor vital signs. No further treatment is necessary, pain should resolve spontaneously within a few minutes. In case of further need of UCA in these patients, an alternative such as SonoVue or Optison may be used [48].

### Responding to adverse events

Once an allergic reaction is identified, the patient should be assessed, and treatment should commence based on the symptoms, and the supervising doctor should immediately be notified. Depending on the severity of the reaction, the cardiopulmonary resuscitation team may be required [1].

Even though life-threatening reactions are exceedingly rare, it is recommended that centres using UCA have a policy in place to enable early detection and rapid response to reactions. All personnel directly involved with administering UCA should be able to identify an allergic reaction and be familiar with the appropriate treatment. Allergy kits should be available and easily accessible in all areas where UCA is administered. These kits contain: auto-injectable epinephrine, hydrocortisone and chlorphenamine. These kits should be maintained and checked regularly for expiry dates. Cardiopulmonary resuscitation personnel and equipment should be readily available before UCA administration [16]. Most adverse reactions are observed soon after administration of UCA [49]. The BSE recommends that patients remain in echo departments for approximately 10 min after administration of UCA to ensure that timely assistance can be provided in the unlikely event of an adverse event. Patients should also be warned about the remote possibility of a delayed reaction [49].

## Training and accreditation requirements

### Key points

1. Staff involved in contrast echocardiography should receive training in all aspects of the procedure.
2. Adequate knowledge should be demonstrated, and competency assessed.
3. At least one member of the contrast team should be ILS trained.
4. Every effort should be made to facilitate the administration of contrast where indicated. This is easily achieved through a systematic team approach between echocardiographers, doctors, and nurses. The role of each will vary depending on individual departments standard operating procedures.

All personnel involved in contrast echocardiography require relevant training. Training should include microbubble physics, instrumentation, and the application of cardiac ultrasound scanning techniques for the acquisition of high-quality images. Knowledge of the clinical indications for the use of UCA is essential and once the need for administration has been identified members of the team should be able to explain the procedure to the patient, including rationale, contraindications and risks and obtain consent (verbal or written). Once UCA has been administered, members should be able to identify side effects and how to manage and report these events, an additional role of monitoring patients who develop side effects may be required. Echocardiographers able to use a PSD to administer UCA must undergo training and obtain certification in intravenous cannulation and drug administration as well as immediate life support (ILS).

UCA- specific product knowledge and injection techniques are essential, this is routinely provided by manufacturers/distributors of commercially available UCA. The BSE is committed to providing future training through distance learning modules via the BSE E-Learning platform.

Not only should all personnel involved in contrast echocardiography be adequately trained, but they should also demonstrate competency. As a minimum standard, echocardiographers should hold BSE accreditation in adult TTE (or equivalent) and have adequate experience in TTE. Beyond the basic requirement of TTE accreditation, the use of UCA in resting or stress echocardiography, or both, requires a level of experience obtained through exposure and performance, initially with guidance and supervision. Echocardiographers are encouraged to pursue courses, tutorials, and preceptorships to learn the appropriate techniques for administering UCA and interpreting contrast-enhanced echocardiograms [16].

At the time of writing, the BSE accreditation process does not assess the adequate and/or appropriate use of UCA during routine echocardiography. It is therefore recommended that individual departments should establish local competency measures. This should be guided by hospital policy and include requirements relating to training, supervision, credentials/qualifications, and experience required. Appendix E provides an example of a local competency assessment tool that can be adapted.

## Successful implementation of UCA guidelines—a team approach

The clinical value of using UCA during echocardiography is incontrovertible. Every effort should be made to facilitate the administration on UCA where indicated. This is easily achieved through a systematic team approach between echocardiographers, nurses and doctors. The most efficient way to integrate UCA use into echocardiography practice is when echocardiographers can independently identify patients who require UCA as part of a study, insert IV cannula and administer.

However, similar level of efficiency can be achieved in three different ways, (1) an appropriately trained healthcare professional (echocardiographer, cardiac physiologist, cardiac scientist, healthcare assistant, nurse) inserts an IV cannula and administers UCA with the use of a PSD, (2) a nurse inserts an IV cannula and administers UCA under a PGD (unless an independent prescriber), (3) a doctor inserts an IV cannula and administers UCA. Echocardiographers should never administer UCA in isolation, another member of staff should always be present, both for assistance during administration and in case of a serious adverse event. Ideally, an experienced practitioner should be available to guide echocardiographers in obtaining appropriate contrast images, if required, and to answer clinical questions.

To ensure safe and efficient practice, a written operating procedure/protocol should set out the indications, injection, and imaging protocols (including the use of a PSD/PGD if required), and personnel responsibilities.

## Conclusion

Contrast echocardiography is effective in improving assessment of cardiac structure and function both at rest and during stress echocardiography, its safety is well documented. Thus, the use of UCA during routine echocardiography should be encouraged through establishment of protocols, training and through setting up the infrastructure for seamless and safe administration. The use of UCA to facilitate echocardiography where indicated should become routine practice in every echocardiography department.

### Supplementary Information

**Additional file 1. Video 1** - Normal flash replenishment of contrast in the myocardium for perfusion assessment.

**Additional file 2. Video 2** - Abnormal flash replenishment of contrast in the myocardium for perfusion assessment.

**Additional file 3. Video 3** - Normal myocardial perfusion in the presence of abnormal wall thickening, as seen in LBBB.

**Additional file 4. Video 4** - Swirling artefact.

**Additional file 5. Video 5** - Attenuation artefact.

**Additional file 6. Video 6** - Blooming artefact.

**Additional file 7. Video 7** - Combination of artefact.

**Additional file 8. Video 8** - Rib/papillary muscle artefact.

## Data Availability

Not applicable.

## Appendix A: Contrast specific imaging techniques and practical applications for the Philips and GE systems

**Table:**

Philips	Name of imaging software	Probe	Transmit/ receive frequency	Cancelation sequence	Effect ( ±)	Mode	MI
*Default settings*							
EPIQ CVx Affinity iE33	LVO (CGen/CRes)	S5-1 X5-1	1.6/3.2 MHz 1.3/2.6 MHz	Power modulation	( +) High temporal & spatial resolution (−) Higher MI mode results in contrast destruction and apical swirling (−) Basal artefacts due to less far field harmonics ( +) Lower transmit frequency allows better penetration	Harmonic	> 0.2 (Intermediate)
EPIQ CVx Affinity iE33	LVO (Cpen)	S5-1 X5-1	2.0/2.0 MHz 1.6/1.6 MHz	Power modulation	( +) Fewer basal artefacts (−) Lower spatial resolution ( +) Lower transmit frequency allows better penetration	Fundamental	< 0.2 (Low)
EPIQ CVx iE33	Low MI (CPen)	S5-1 X5-1	2.0/2.0 MHz 1.6/1.6 MHz	Power modulation (Additional optimisation through Xres settings, maps etc.)	( +) Uniform LV opacification & fewer artefacts such as apical swirling ( +) Improves signal to noise ratio ( +) Simultaneous assessment of myocardial perfusion because of improved cancellation technique ( +) Minimal contrast destruction ( +) Generally sharper image than LVO (−) Low temporal and spatial resolution ( +) Lower transmit frequency allows better penetration	Fundamental	< 0.2 (Low)

General Electric (GE)	Name of imaging software	Probe	Transmit/ receive frequency	Cancelation sequence	Effect ( ±)	Mode	MI
*Default settings*							
Vivid E95	LV Contrast	4Vc-D M5Sc-D	1.7/3.3 MHz 1.4/2.8 MHz	Pulse inversion	( +) High resolution ( +) Excellent tissue signal suppression (−) Higher MI result in contrast destruction and apical swirling (−) Basal attenuation due to filtering at higher frequencies ( +) Lower transmit frequency allows better penetration	Harmonic	> 0.2 (Intermediate)
Vivid E95	LVO Stress	4Vc-D M5Sc-D	1.7/3.3 MHz 1.4/2.8 MHz	Pulse inversion	( +) High resolution ( +) Excellent tissue signal suppression (−) Higher MI result in contrast destruction and apical swirling (-) Basal attenuation due to filtering at higher frequencies ( +) Lower transmit frequency allows better penetration	Harmonic	> 0.2 (Intermediate)
Vivid E95	Contrast Low MI	4Vc-D M5Sc-D	1.7/3.3 MHz 1.4/2.8 MHz	Pulse inversion	At present not optimised. Work in progress	Harmonic	< 0.2 (Low)

Symbols in text + advantage, − disadvantage

## Appendix B: Optimal low MI settings based on the EPIQ CVx 7.0.3 with a 2D probe

**Table:**

Variable	Setting
Gain	61%
Depth	15 cm
Frequency	25 Hz
Compression	50
Persistence	Low
CPEN	CPEN
Chroma Map	Pink
Gray Map	2
XRES	ON
Output Power	20.5db/MI 0.10
Flash Power	5.5db/ MI 0.63F
Flash frames	5
Loop Length	10 beats
2D PRF	High
Res/Spd	12 o’clock

## Appendix C: How to perform contrast echocardiography for perfusion assessment

Low MI contrast specific imaging mode should be selected, the time gain compensation (TGC) should be set so that myocardial and LV opacification appear uniform from apex to base. Typically, this requires moving the near field TGC depending on the type of equipment to either upwards or to the right. The focus should be set at the mitral valve level. The overall 2D gains should be maintained at around 65%. Background gains are set so that minimal tissue signal is seen. This is best achieved in the Philips system by pressing the “iscan” button and in the GE system the “auto” button. Following these adjustments, UCA is administered intravenously at a constant infusion rate with the use of an infusion pump connected to a dedicated intravenous line (Vuejet pump). The infusion rate will vary according to the product used. For Sonovue the initial dose is 1 mL/min. For Luminity or Optison it is 0.3–0.5 mL/min. Imaging is performed in the three apical views. Following 1 min of the infusion when steady state in the circulation is usually obtained, imaging should begin. The infusion rate should be adjusted to obtain the best possible myocardial opacification with minimal attenuation. Care should also be taken to avoid oversaturating the field of view with contrast (signal intensity should be lower in the myocardium compared with the LV cavity). Once optimized, the machine settings should be kept constant throughout the study. The focus maybe moved towards the apex when an apical perfusion defect is seen to eliminate near field bubble destruction artefact. Nonstandard apical views (e.g., bringing the lateral wall into the sector field) maybe used to overcome basal attenuation artefacts. In large left ventricles, each myocardial wall (i.e., inferior, and anterior walls) maybe imaged separately when artefacts are observed in the peripheral fields. Commence flash-replenishment imaging by pressing the “Flash” button which transiently deliver ultrasound waves at high MI (> 0.6 < 0.9) to clear myocardial microbubbles. Begin with 5 flash frames, if it fails to clear completely as observed by residual myocardial signal then increase the number to 10 then to 20 frames—if still not successful then increase the flash MI to 0.9. Also care needs to be taken not to clear microbubbles from the LV cavity as it will delay appearance of microbubble in the myocardium due to delay of transit from the pre-capillary level to the capillary level, which can affect perfusion evaluation. Following flash, acquire 10–15 cardiac cycles (10 s) both in real-time and in triggered end-systolic mode in each view reverting to low MI imaging. The same sequence should be followed at peak stress with no change in settings. However, during hyperaemia, with increase in capillary recruitment due to increased myocardial oxygen consumption, the microbubble concentration may exceed the dynamic range of the machine. This will result in failure to discern perfusion defect. Thus, when myocardial opacification intensity is similar or exceed that of LV opacification reduce the infusion rate till myocardial opacification intensity is less than LV opacification. Slow bolus doses may also be used (Sonovue or Optison 0.4 to 0.5 mL, followed by 5 mL of normal saline over 20 s) or Luminity 0.2 mL diluted 1 mL normal saline over 20 s. However, with bolus quantification may not be as robust. For treadmill or bicycle exercise, administer bolus UCA approximately 10 secs (Sonovue) or 20secs (Luminity) before termination of stress and begin the acquisition of images. At the same time commence UCA infusion. Acquire perfusion images soon after wall motion loops are acquired.

### Interpretation of perfusion

Myocardial perfusion should be assessed at end-systole. The capillaries are most prominent at end-systole-arterioles empty blood into the arteries and venules into capillaries in systole. At rest normal myocardial opacification is considered if the myocardial bed opacifies homogenously by 5 secs after flash-grade 2, grade 1 if heterogenous opacification occurs after 5 s; and 0 if there is no opacification after 10 s [3, 38]. This gives an assessment of myocardial viability in dysfunctional myocardium—Grade 2 suggests good myocardial viability and grade 0, no myocardial viability. Grade 1 intermediate probability of myocardial viability. These scores are assigned to each of the evaluable myocardial segments in the standard 17 segment model. Perfusion abnormality during stress is defined as replenishment of the myocardial segments beyond 2 s after flash at peak stress accompanied by subendocardial defect or transmural defect replenishing from subepicardium to subendocardium.
